# Renal replacement therapy does have impact on beta-lactam clearance

**DOI:** 10.1186/s13613-023-01148-3

**Published:** 2023-06-06

**Authors:** Zhenwei Yu

**Affiliations:** Sir Run Run Shaw Hospital, School of Medicine, Zhejiang University, 3Rd East Qingchun Road, Handzhou, China

Dear editor,

Roux et al. had published a study recently which concluded that renal replacement therapy (RRT) initiation strategy did not significantly influence plasma trough concentrations of beta-lactams in ICU patients with severe AKI [1]. I want to add some comments.

## Most beta-lactams can be eliminated by RRT

Beta-lactams have similar chemical structures and thus have similar in vivo behaviors. Previous studies have shown that most beta-lactams can be eliminated by RRT at different extents. The clearance of RRT contributed to the total clearance of drugs. For some beta-lactams, such as cefotaxime the most frequent dosed beta-lactam in Roux, et al.’s study, the clearance of continuous RRT was comparable to normal renal clearance [2]. When the patients were suffering acute kidney injury (AKI), RRT would be the main elimination route for most beta-lactams and the influence of RRT should be taken into serious consideration. Numerous studies had proved the influence of RRT on removal of beta-lactams and suggested dosing for patients receiving RRT [3].

## Beta-lactam plasma concentrations were influenced by multiple factors

Although RRT contributed to elimination of beta-lactams, the plasma concentrations were influenced by multiple factors. RRT can lower the concentration of beta-lactams, but the decreased renal clearance due to AKI in these patients can elevate the concentrations. Common physio-pathological conditions in critically ill patients, such as sepsis and hypoproteinemia, would alter the distribution volume and clearance, thus have a complicated impact on the concentrations [4].

Finally, the dose of beta-lactams is a critical factor we can control and it was associated with plasma concentrations and pharmacokinetic/pharmacodynamic (PK/PD) target attainment. Most beta-lactams were suggested to decrease dose for patient with impaired renal function, whether chronic or acute. However, the normalized dose of beta-lactams per 24 h in patients with adequate through concentration in Roux’s study were higher than 1. It is unusual to give AKI patients a beta-lactams dose larger than suggested to patient with normal renal function. It would be better if Roux’s study presents the detailed through concentrations of beta-lactams, rather than target attainments only. There is, in addition, one further point to make. It is inappropriate for Roux et al. to conclude that RRT initiation strategy did not significantly influence plasma trough concentrations, as the authors only found that there is no difference in target attainments.

## Dosing of beta-lactams, do not only focus on antibacterial effects

The PK/PD index associated with efficacy of beta-lactams is fraction time of concentration above minimum inhibitory concentrations (*f*T > MIC). For critic ill patients, the *f*T > 4 × MIC should be maintained during the whole dosing interval, and this target was also used in Roux’s study. This is the low limit of beta-lactam concentrations to assure the efficacy; however, the up limit to avoid toxicity is lacking of data support and always neglected in studies. Although beta-lactams have good safety profiles, overdose of beta-lactams would cause severe adverse effects, or even related to a high mortality [5]. I concerned about the safety profile of patients receiving beta-lactam regimen above the regular regimen recommended in stable patients. The physicians also struggle in clinical practice when dosing beta-lactams for critic ill patient receiving RRT and with AKI. Studies are urgently needed to recommend an empiric dose regimen for these patients. Due to the complexity of condition of critically ill patients, therapeutic drug monitoring would be a powerful tool to optimize the dosing of beta-lactams.

## Data Availability

Not applicable.
